# Supplementary material for: End user experiences of an electronic health records platform in a tertiary hospital system in Kenya
Source: PLoS One. 2025 Sep 19;20(9):e0332249. doi: 10.1371/journal.pone.0332249 (PMC12449015; doi:10.1371/journal.pone.0332249)
Supplement: S1 Table — (DOCX) [file pone.0332249.s001.docx]

**SUPPLEMENTARY TABLES**

|  | | Gender | | | | | | P Value |
| --- | --- | --- | --- | --- | --- | --- | --- | --- |
|  |  | Males | | | Females | | |  |
|  |  | (n = 199) | | | (n = 272) | | |  |
| Current Age (years) | < 30 years | 13 | 6.60% | 16 | | 5.90% | 0.392 | |
|  | 30 - 39 years | 95 | 48.20% | 120 | | 44.10% |  |  |
|  | 40 - 49 years | 65 | 33.00% | 106 | | 39.00% |  |  |
|  | 50 - 59 years | 22 | 11.20% | 30 | | 11.00% |  |  |
|  | >= 60 years | 2 | 1.00% | 0 | | 0.00% |  |  |
| **EXPERIENCE WITH EHR** | | | | | | | | |
| How long have you been using the EHR at AKUHN (months) | < 6 months | 18 | 9.0% | 32 | | 11.8% | 0.368 | |
|  | >= 6 months | 181 | 91.0% | 240 | | 88.2% |  |  |
| How has the use of the EHR changed your work compared to using a paper-based medical record system? | Much Worse / Worse | 8 | 4.06% | 12 | | 4.44% | 0.800 | |
|  | Neither worse nor better | 13 | 6.60% | 14 | | 5.19% |  |  |
|  | Much Better / Better | 176 | 89.34% | 244 | | 90.37% |  |  |
| Prior to using this EHR at AKUHN, have you used another EHR system? | Yes | 52 | 26.10% | 50 | | 18.50% | 0.054 | |
|  | No | 147 | 73.90% | 220 | | 81.50% |  |  |
| How would you rate this EHR at AKUHN as compared to other EHR systems you used in the past? | Much Worse / Worse | 3 | 5.77% | 2 | | 4.00% | 0.511 | |
|  | Neither worse nor better | 3 | 5.77% | 6 | | 12.00% |  |  |
|  | Much Better / Better | 46 | 88.46% | 42 | | 84.00% |  |  |
| **EHR USE ENVIRONMENT** | | | | | | | | |
| I received adequate training on how to use this EHR | Strongly disagree | 1 | 0.50% | 6 | | 2.30% | 0.207 | |
|  | Neither agree nor disagree | 7 | 3.60% | 5 | | 1.90% |  |  |
|  | Strongly agree | 187 | 95.90% | 254 | | 95.80% |  |  |
| My questions about use of this EHR were sufficiently answered | Strongly disagree | 2 | 1.00% | 3 | | 1.10% | 0.655 | |
|  | Neither agree nor disagree | 11 | 5.60% | 10 | | 3.80% |  |  |
|  | Strongly agree | 183 | 93.40% | 251 | | 95.10% |  |  |
| I receive technical support whenever I need it | Strongly disagree | 5 | 2.60% | 5 | | 1.90% | 0.568 | |
|  | Neither agree nor disagree | 12 | 6.10% | 11 | | 4.20% |  |  |
|  | Strongly agree | 179 | 91.30% | 248 | | 93.90% |  |  |
| I am satisfied with the technical support I have received in use of this EHR | Strongly disagree | 4 | 2.00% | 6 | | 2.30% | 0.143 | |
|  | Neither agree nor disagree | 22 | 11.20% | 16 | | 6.00% |  |  |
|  | Strongly agree | 171 | 86.80% | 243 | | 91.70% |  |  |
| The system downtimes are acceptable | Strongly disagree | 14 | 7.10% | 14 | | 5.30% | 0.168 | |
|  | Neither agree nor disagree | 66 | 33.70% | 111 | | 42.00% |  |  |
|  | Strongly agree | 116 | 59.20% | 139 | | 52.70% |  |  |
| When this EHR system is down, we have policies and procedures to allow the clinician to continue to see patients | Strongly disagree | 112 | 57.10% | 142 | | 53.80% | 0.745 | |
|  | Neither agree nor disagree | 32 | 16.30% | 49 | | 18.60% |  |  |
|  | Strongly agree | 52 | 26.50% | 73 | | 27.70% |  |  |
| The EHR screens respond to my actions instantly | Strongly disagree | 13 | 6.60% | 24 | | 9.10% | 0.002 | |
|  | Neither agree nor disagree | 63 | 32.00% | 47 | | 17.70% |  |  |
|  | Strongly agree | 121 | 61.40% | 194 | | 73.20% |  |  |
| Our facility has adequate computer terminals to access this EHR | Strongly disagree | 18 | 9.20% | 17 | | 6.50% | 0.024 | |
|  | Neither agree nor disagree | 44 | 22.40% | 37 | | 14.10% |  |  |
|  | Strongly agree | 134 | 68.40% | 209 | | 79.50% |  |  |
| Lab results appear in this EHR in a timely fashion | Strongly disagree | 47 | 24.70% | 56 | | 21.90% | 0.759 | |
|  | Neither agree nor disagree | 65 | 34.20% | 89 | | 34.80% |  |  |
|  | Strongly agree | 78 | 41.10% | 111 | | 43.40% |  |  |
| Radiology results appear in this EHR in a timely fashion | Strongly disagree | 47 | 24.40% | 63 | | 24.70% | 0.787 | |
|  | Neither agree nor disagree | 70 | 36.30% | 85 | | 33.30% |  |  |
|  | Strongly agree | 76 | 39.40% | 107 | | 42.00% |  |  |
| This EHR allows me to review trends in lab values | Strongly disagree | 17 | 8.90% | 6 | | 2.40% | 0.002 | |
|  | Neither agree nor disagree | 27 | 14.10% | 24 | | 9.40% |  |  |
|  | Strongly agree | 147 | 77.00% | 225 | | 88.20% |  |  |
| The project plan was adequately communicated to us during implementation | Strongly disagree | 7 | 3.70% | 8 | | 3.10% | 0.494 | |
|  | Neither agree nor disagree | 27 | 14.10% | 28 | | 10.70% |  |  |
|  | Strongly agree | 157 | 82.20% | 226 | | 86.30% |  |  |
| Adequate resources were committed to the implementation | Strongly disagree | 8 | 4.20% | 6 | | 2.30% | 0.450 | |
|  | Neither agree nor disagree | 22 | 11.50% | 26 | | 10.10% |  |  |
|  | Strongly agree | 162 | 84.40% | 225 | | 87.50% |  |  |
| What is the main device you use to access the EHR? | Desktop Computer | 177 | 88.90% | 248 | | 91.20% | 0.378 | |
|  | Laptop | 67 | 33.70% | 73 | | 26.80% |  |  |
|  | Tablet | 3 | 1.50% | 8 | | 2.90% |  |  |
|  | Phone | 8 | 4.00% | 8 | | 2.90% |  |  |
| To see patient test results | Never | 175 | 90.70% | 243 | | 91.70% | 0.689 | |
|  | Occasionally | 12 | 6.20% | 12 | | 4.50% |  |  |
|  | Frequently / Always | 6 | 3.10% | 10 | | 3.80% |  |  |
| To obtain contact information for a patient | Never | 173 | 89.60% | 244 | | 92.40% | 0.537 | |
|  | Occasionally | 12 | 6.20% | 13 | | 4.90% |  |  |
|  | Frequently / Always | 8 | 4.10% | 7 | | 2.70% |  |  |
| To document something in the patient medical record | Never | 173 | 89.60% | 234 | | 88.60% | 0.790 | |
|  | Occasionally | 13 | 6.70% | 22 | | 8.30% |  |  |
|  | Frequently / Always | 7 | 3.60% | 8 | | 3.00% |  |  |
| To identify patients with gaps in their medical care | Never | 176 | 90.70% | 240 | | 92.00% | 0.869 | |
|  | Occasionally | 12 | 6.20% | 13 | | 5.00% |  |  |
|  | Frequently / Always | 6 | 3.10% | 8 | | 3.10% |  |  |
| Do you use paper records for any other reason apart from mentioned above? | Yes | 25 | 13.30% | 19 | | 7.30% | 0.037 | |
|  | No | 163 | 86.70% | 242 | | 92.70% |  |  |
| **EHR IMPACT** | | | | | | | | |
| Costs to my practice in providing care | Highly detrimental / detrimental | 15 | 8.20% | 11 | | 4.40% | 0.243 | |
|  | Neither detrimental nor beneficial | 13 | 7.10% | 17 | | 6.70% |  |  |
|  | Highly beneficial / beneficial | 154 | 84.60% | 224 | | 88.90% |  |  |
| Autonomy of healthcare providers | Highly detrimental / detrimental | 8 | 4.30% | 8 | | 3.10% | 0.233 | |
|  | Neither detrimental nor beneficial | 14 | 7.50% | 31 | | 12.10% |  |  |
|  | Highly beneficial / beneficial | 165 | 88.20% | 217 | | 84.80% |  |  |
| Quality of health care | Highly detrimental / detrimental | 1 | 0.50% | 1 | | 0.40% | 0.448 | |
|  | Neither detrimental nor beneficial | 10 | 5.30% | 8 | | 3.10% |  |  |
|  | Highly beneficial / beneficial | 176 | 94.10% | 248 | | 96.50% |  |  |
| Interactions within the healthcare team | Highly detrimental / detrimental | 12 | 6.30% | 21 | | 8.20% | 0.603 | |
|  | Neither detrimental nor beneficial | 23 | 12.20% | 37 | | 14.40% |  |  |
|  | Highly beneficial / beneficial | 154 | 81.50% | 199 | | 77.40% |  |  |
| Enjoyment of clinical practice | Highly detrimental / detrimental | 10 | 5.30% | 21 | | 8.20% | 0.261 | |
|  | Neither detrimental nor beneficial | 20 | 10.50% | 35 | | 13.70% |  |  |
|  | Highly beneficial / beneficial | 160 | 84.20% | 200 | | 78.10% |  |  |
| Stress levels of healthcare providers | Highly detrimental / detrimental | 19 | 10.00% | 35 | | 13.60% | 0.175 | |
|  | Neither detrimental nor beneficial | 20 | 10.50% | 38 | | 14.80% |  |  |
|  | Highly beneficial / beneficial | 151 | 79.50% | 184 | | 71.60% |  |  |
| Healthcare providers self-image | Highly detrimental / detrimental | 14 | 7.40% | 31 | | 12.10% | 0.184 | |
|  | Neither detrimental nor beneficial | 23 | 12.20% | 38 | | 14.80% |  |  |
|  | Highly beneficial / beneficial | 151 | 80.30% | 188 | | 73.20% |  |  |
| Humaneness of clinical practice | Highly detrimental / detrimental | 17 | 8.90% | 27 | | 10.50% | 0.786 | |
|  | Neither detrimental nor beneficial | 29 | 15.30% | 43 | | 16.70% |  |  |
|  | Highly beneficial / beneficial | 144 | 75.80% | 187 | | 72.80% |  |  |
| The rapport between healthcare providers and patients | Highly detrimental / detrimental | 13 | 6.90% | 28 | | 10.90% | 0.359 | |
|  | Neither detrimental nor beneficial | 27 | 14.30% | 35 | | 13.70% |  |  |
|  | Highly beneficial / beneficial | 149 | 78.80% | 193 | | 75.40% |  |  |
| Personal and professional privacy | Highly detrimental / detrimental | 10 | 5.30% | 15 | | 5.80% | 0.896 | |
|  | Neither detrimental nor beneficial | 16 | 8.40% | 24 | | 9.30% |  |  |
|  | Highly beneficial / beneficial | 164 | 86.30% | 218 | | 84.80% |  |  |
| Healthcare providers access to up to-date information | Highly detrimental / detrimental | 5 | 2.60% | 5 | | 1.90% | 0.709 | |
|  | Neither detrimental nor beneficial | 11 | 5.80% | 19 | | 7.40% |  |  |
|  | Highly beneficial / beneficial | 174 | 91.60% | 233 | | 90.70% |  |  |
| Patients satisfaction with the quality of care they receive | Highly detrimental / detrimental | 6 | 3.20% | 3 | | 1.20% | 0.298 | |
|  | Neither detrimental nor beneficial | 21 | 11.10% | 25 | | 9.80% |  |  |
|  | Highly beneficial / beneficial | 162 | 85.70% | 227 | | 89.00% |  |  |
| Generalists ability to manage more complex problems | Highly detrimental / detrimental | 3 | 1.60% | 7 | | 2.70% | 0.516 | |
|  | Neither detrimental nor beneficial | 23 | 12.20% | 24 | | 9.40% |  |  |
|  | Highly beneficial / beneficial | 163 | 86.20% | 225 | | 87.90% |  |  |
| Comprehensiveness of patient care | Highly detrimental / detrimental | 2 | 1.10% | 9 | | 3.50% | 0.105 | |
|  | Neither detrimental nor beneficial | 16 | 8.40% | 13 | | 5.10% |  |  |
|  | Highly beneficial / beneficial | 172 | 90.50% | 235 | | 91.40% |  |  |
| Efficiency of clinical practice | Highly detrimental / detrimental | 3 | 1.60% | 7 | | 2.70% | 0.574 | |
|  | Neither detrimental nor beneficial | 12 | 6.30% | 12 | | 4.70% |  |  |
|  | Highly beneficial / beneficial | 175 | 92.10% | 238 | | 92.60% |  |  |
| Avoiding errors (such as overlooking drug interactions) | Highly detrimental / detrimental | 7 | 3.70% | 12 | | 4.70% | 0.844 | |
|  | Neither detrimental nor beneficial | 12 | 6.30% | 18 | | 7.00% |  |  |
|  | Highly beneficial / beneficial | 171 | 90.00% | 227 | | 88.30% |  |  |
| **EHR FUNCTIONALITY** | | | | | | | | |
| Obtain and review patient information and data | Very difficult / Somewhat difficult | 3 | 1.60% | 4 | | 1.60% | 0.712 | |
|  | No difference | 4 | 2.10% | 3 | | 1.20% |  |  |
|  | Very easy / Somewhat easy | 182 | 96.30% | 246 | | 97.20% |  |  |
| Document care for my patients | Very difficult / Somewhat difficult | 3 | 1.60% | 4 | | 1.60% | 0.387 | |
|  | No difference | 2 | 1.10% | 0 | | 0.00% |  |  |
|  | Very easy / Somewhat easy | 183 | 97.30% | 244 | | 98.40% |  |  |
| View lab results for my patients | Very difficult / Somewhat difficult | 2 | 1.10% | 2 | | 0.80% | 0.535 | |
|  | No difference | 3 | 1.60% | 1 | | 0.40% |  |  |
|  | Very easy / Somewhat easy | 182 | 97.30% | 246 | | 98.80% |  |  |
| View radiology results for my patients | Very difficult / Somewhat difficult | 2 | 1.10% | 2 | | 0.80% | 0.714 | |
|  | No difference | 4 | 2.10% | 3 | | 1.20% |  |  |
|  | Very easy / Somewhat easy | 181 | 96.80% | 242 | | 98.00% |  |  |
| Prevent adverse events (e.g, drug-drug interaction, drug-allergy interaction) | Very difficult / Somewhat difficult | 3 | 1.60% | 5 | | 2.10% | 0.573 | |
|  | No difference | 6 | 3.20% | 4 | | 1.60% |  |  |
|  | Very easy / Somewhat easy | 178 | 95.20% | 234 | | 96.30% |  |  |
| Track preventive care for my patients | Very difficult / Somewhat difficult | 2 | 1.10% | 4 | | 1.60% | 0.380 | |
|  | No difference | 11 | 6.00% | 8 | | 3.20% |  |  |
|  | Very easy / Somewhat easy | 171 | 92.90% | 236 | | 95.20% |  |  |
| Manage chronic disease conditions for my patients | Very difficult / Somewhat difficult | 1 | 0.50% | 3 | | 1.20% | 0.459 | |
|  | No difference | 11 | 5.90% | 9 | | 3.60% |  |  |
|  | Very easy / Somewhat easy | 173 | 93.50% | 236 | | 95.20% |  |  |
| Manage orders | Very difficult / Somewhat difficult | 2 | 1.10% | 7 | | 2.80% | 0.004 | |
|  | No difference | 6 | 3.20% | 0 | | 0.00% |  |  |
|  | Very easy / Somewhat easy | 177 | 95.70% | 242 | | 97.20% |  |  |
| Manage referrals | Very difficult / Somewhat difficult | 3 | 1.60% | 4 | | 1.60% | 1.000 | |
|  | No difference | 4 | 2.20% | 5 | | 2.00% |  |  |
|  | Very easy / Somewhat easy | 176 | 96.20% | 240 | | 96.40% |  |  |
| Provide patient educational materials | Very difficult / Somewhat difficult | 4 | 2.20% | 4 | | 1.60% | 0.726 | |
|  | No difference | 13 | 7.10% | 13 | | 5.30% |  |  |
|  | Very easy / Somewhat easy | 167 | 90.80% | 226 | | 93.00% |  |  |
| Analyze outcomes of care | Very difficult / Somewhat difficult | 4 | 2.20% | 3 | | 1.20% | 0.241 | |
|  | No difference | 8 | 4.40% | 5 | | 2.00% |  |  |
|  | Very easy / Somewhat easy | 171 | 93.40% | 239 | | 96.80% |  |  |
| Access the EHR from offsite locations | Very difficult / Somewhat difficult | 3 | 1.60% | 3 | | 1.20% | 1.000 | |
|  | No difference | 1 | 0.50% | 2 | | 0.80% |  |  |
|  | Very easy / Somewhat easy | 180 | 97.80% | 238 | | 97.90% |  |  |
| Communicate with my colleagues to coordinate care | Very difficult / Somewhat difficult | 2 | 1.10% | 8 | | 3.20% | 0.249 | |
|  | No difference | 11 | 6.00% | 10 | | 4.00% |  |  |
|  | Very easy / Somewhat easy | 170 | 92.90% | 231 | | 92.80% |  |  |
| Communicate with my patients | Very difficult / Somewhat difficult | 3 | 1.70% | 7 | | 2.90% | 0.693 | |
|  | No difference | 12 | 6.70% | 14 | | 5.80% |  |  |
|  | Very easy / Somewhat easy | 164 | 91.60% | 221 | | 91.30% |  |  |
| Enhance the continuity of care the hospital can provide | Very difficult / Somewhat difficult | 2 | 1.10% | 5 | | 2.00% | 0.558 | |
|  | No difference | 6 | 3.30% | 5 | | 2.00% |  |  |
|  | Very easy / Somewhat easy | 175 | 95.60% | 235 | | 95.90% |  |  |
| Are there any functionalities missing in this EHR? | No | 173 | 92.00% | 238 | | 93.30% | 0.711 | |
|  | Yes | 15 | 8.00% | 17 | | 6.70% |  |  |
| Is there any additional help you would need to use the EHR? | No | 178 | 95.20% | 247 | | 95.70% | 0.819 | |
|  | Yes | 9 | 4.80% | 11 | | 4.30% |  |  |
| **OVERALL EVALUATION OF THE EHR** | | | | | | | | |
| To me, use of this EHR is easy | Strongly disagree | 4 | 2.10% | 2 | | 0.80% | 0.515 | |
|  | Neither agree nor disagree | 10 | 5.20% | 12 | | 4.60% |  |  |
|  | Strongly agree | 178 | 92.70% | 245 | | 94.60% |  |  |
| The EHR screens are user friendly | Strongly disagree | 7 | 3.60% | 6 | | 2.30% | 0.651 | |
|  | Neither agree nor disagree | 10 | 5.20% | 12 | | 4.60% |  |  |
|  | Strongly agree | 175 | 91.10% | 244 | | 93.10% |  |  |
| This EHR provides all functionalities that I expect | Strongly disagree | 7 | 3.70% | 7 | | 2.70% | 0.726 | |
|  | Neither agree nor disagree | 12 | 6.30% | 20 | | 7.60% |  |  |
|  | Strongly agree | 172 | 90.10% | 236 | | 89.70% |  |  |
| Overall, I am satisfied with my experience with this EHR | Strongly disagree | 6 | 3.10% | 5 | | 1.90% | 0.736 | |
|  | Neither agree nor disagree | 19 | 9.80% | 26 | | 9.80% |  |  |
|  | Strongly agree | 168 | 87.00% | 233 | | 88.30% |  |  |
| I would recommend this EHR to other similar practices | Strongly disagree | 6 | 3.10% | 9 | | 3.40% | 0.072 | |
|  | Neither agree nor disagree | 37 | 19.30% | 30 | | 11.50% |  |  |
|  | Strongly agree | 149 | 77.60% | 223 | | 85.10% |  |  |
| My colleagues have negative opinions about this EHR | Strongly disagree | 97 | 50.30% | 174 | | 65.90% | <0.001 | |
|  | Neither agree nor disagree | 75 | 38.90% | 58 | | 22.00% |  |  |
|  | Strongly agree | 21 | 10.90% | 32 | | 12.10% |  |  |
| Use of this EHR interferes with my work | Strongly disagree | 159 | 82.40% | 226 | | 85.90% | 0.189 | |
|  | Neither agree nor disagree | 15 | 7.80% | 10 | | 3.80% |  |  |
|  | Strongly agree | 19 | 9.80% | 27 | | 10.30% |  |  |
| I would be in favor of ceasing use of this EHR in our practice | Strongly disagree | 164 | 84.50% | 230 | | 88.10% | 0.221 | |
|  | Neither agree nor disagree | 9 | 4.60% | 5 | | 1.90% |  |  |
|  | Strongly agree | 21 | 10.80% | 26 | | 10.00% |  |  |
| Use of this EHR requires me to do more work compared to what I used to do | Strongly disagree | 156 | 80.40% | 221 | | 83.70% | 0.454 | |
|  | Neither agree nor disagree | 12 | 6.20% | 10 | | 3.80% |  |  |
|  | Strongly agree | 26 | 13.40% | 33 | | 12.50% |  |  |

**Supplementary Table 1: Differences of EHR experiences among males and females.**

|  | | Healthcare Category | | | | | | | | P Value | |
| --- | --- | --- | --- | --- | --- | --- | --- | --- | --- | --- | --- |
|  |  | Allied Health Staff | | Nurses | | | Doctors | | |  | |
|  |  | (n = 122) | | (n = 220) | | | (n = 124) | | |  |  |
| Current Age (years) | < 30 years | 13 | 10.70% | 10 | 4.60% | 6 | | 4.90% | 0.052 | |  |
|  | 30 - 39 years | 61 | 50.00% | 96 | 43.80% | 57 | | 46.30% |  |  |  |
|  | 40 - 49 years | 35 | 28.70% | 92 | 42.00% | 41 | | 33.30% |  |  |  |
|  | >= 50 years | 13 | 10.70% | 21 | 9.60% | 19 | | 15.40% |  |  |  |
| **EXPERIENCE WITH EHR** | | | | | | | | | | | |
| How long have you been using the EHR at AKUHN (months) | < 6 months | 12 | 9.8% | 21 | 9.5% | 17 | | 13.7% | 0.455 | |  |
|  | >= 6 months | 110 | 90.2% | 199 | 90.5% | 107 | | 86.3% |  |  |  |
| How has the use of the EHR changed your work compared to using a paper-based medical record system? | Much Worse / Worse | 10 | 8.40% | 7 | 3.20% | 3 | | 2.42% | <0.001 | |  |
|  | Neither worse nor better | 15 | 12.61% | 3 | 1.37% | 8 | | 6.45% |  |  |  |
|  | Much Better / Better | 94 | 78.99% | 209 | 95.43% | 113 | | 91.13% |  |  |  |
| Prior to using this EHR at AKUHN, have you used another EHR system? | Yes | 28 | 23.00% | 30 | 13.70% | 44 | | 35.50% | <0.001 | |  |
|  | No | 94 | 77.00% | 189 | 86.30% | 80 | | 64.50% |  |  |  |
| How would you rate this EHR at AKUHN as compared to other EHR systems you used in the past? | Much Worse / Worse | 0 | 0.00% | 1 | 3.33% | 4 | | 9.09% | 0.242 | |  |
|  | Neither worse nor better | 2 | 7.14% | 1 | 3.33% | 6 | | 13.64% |  |  |  |
|  | Much Better / Better | 26 | 92.86% | 28 | 93.33% | 34 | | 77.27% |  |  |  |
| **EHR USE ENVIRONMENT** | | | | | | | | | | | |
| I received adequate training on how to use this EHR | Strongly disagree | 2 | 1.70% | 0 | 0.00% | 5 | | 4.10% | 0.005 | |  |
|  | Neither agree nor disagree | 6 | 5.20% | 3 | 1.40% | 3 | | 2.40% |  |  |  |
|  | Strongly agree | 107 | 93.00% | 215 | 98.60% | 115 | | 93.50% |  |  |  |
| My questions about use of this EHR were sufficiently answered | Strongly disagree | 3 | 2.60% | 1 | 0.50% | 1 | | 0.80% | 0.036 | |  |
|  | Neither agree nor disagree | 9 | 7.80% | 5 | 2.30% | 7 | | 5.70% |  |  |  |
|  | Strongly agree | 103 | 89.60% | 212 | 97.20% | 115 | | 93.50% |  |  |  |
| I receive technical support whenever I need it | Strongly disagree | 3 | 2.60% | 2 | 0.90% | 5 | | 4.10% | 0.009 | |  |
|  | Neither agree nor disagree | 12 | 10.40% | 6 | 2.80% | 5 | | 4.10% |  |  |  |
|  | Strongly agree | 100 | 87.00% | 210 | 96.30% | 113 | | 91.90% |  |  |  |
| I am satisfied with the technical support I have received in use of this EHR | Strongly disagree | 4 | 3.40% | 1 | 0.50% | 5 | | 4.10% | <0.001 | |  |
|  | Neither agree nor disagree | 21 | 18.10% | 10 | 4.60% | 7 | | 5.70% |  |  |  |
|  | Strongly agree | 91 | 78.40% | 208 | 95.00% | 111 | | 90.20% |  |  |  |
| The system downtimes are acceptable | Strongly disagree | 10 | 8.70% | 9 | 4.10% | 9 | | 7.30% | 0.134 | |  |
|  | Neither agree nor disagree | 40 | 34.80% | 95 | 43.60% | 40 | | 32.50% |  |  |  |
|  | Strongly agree | 65 | 56.50% | 114 | 52.30% | 74 | | 60.20% |  |  |  |
| When this EHR system is down, we have policies and procedures to allow the clinician to continue to see patients | Strongly disagree | 57 | 49.60% | 136 | 62.10% | 57 | | 46.70% | 0.016 | |  |
|  | Neither agree nor disagree | 20 | 17.40% | 30 | 13.70% | 31 | | 25.40% |  |  |  |
|  | Strongly agree | 38 | 33.00% | 53 | 24.20% | 34 | | 27.90% |  |  |  |
| The EHR screens respond to my actions instantly | Strongly disagree | 10 | 8.60% | 13 | 5.90% | 14 | | 11.40% | 0.481 | |  |
|  | Neither agree nor disagree | 29 | 25.00% | 52 | 23.70% | 27 | | 22.00% |  |  |  |
|  | Strongly agree | 77 | 66.40% | 154 | 70.30% | 82 | | 66.70% |  |  |  |
| Our facility has adequate computer terminals to access this EHR | Strongly disagree | 5 | 4.40% | 14 | 6.40% | 16 | | 13.00% | 0.034 | |  |
|  | Neither agree nor disagree | 22 | 19.30% | 45 | 20.60% | 14 | | 11.40% |  |  |  |
|  | Strongly agree | 87 | 76.30% | 159 | 72.90% | 93 | | 75.60% |  |  |  |
| Lab results appear in this EHR in a timely fashion | Strongly disagree | 22 | 21.00% | 50 | 23.00% | 30 | | 25.00% | 0.061 | |  |
|  | Neither agree nor disagree | 36 | 34.30% | 87 | 40.10% | 30 | | 25.00% |  |  |  |
|  | Strongly agree | 47 | 44.80% | 80 | 36.90% | 60 | | 50.00% |  |  |  |
| Radiology results appear in this EHR in a timely fashion | Strongly disagree | 20 | 19.00% | 55 | 25.20% | 34 | | 28.10% | 0.008 | |  |
|  | Neither agree nor disagree | 41 | 39.00% | 86 | 39.40% | 27 | | 22.30% |  |  |  |
|  | Strongly agree | 44 | 41.90% | 77 | 35.30% | 60 | | 49.60% |  |  |  |
| This EHR allows me to review trends in lab values | Strongly disagree | 6 | 5.70% | 13 | 6.00% | 4 | | 3.40% | 0.001 | |  |
|  | Neither agree nor disagree | 25 | 23.60% | 18 | 8.30% | 8 | | 6.80% |  |  |  |
|  | Strongly agree | 75 | 70.80% | 187 | 85.80% | 106 | | 89.80% |  |  |  |
| The project plan was adequately communicated to us during implementation | Strongly disagree | 3 | 2.70% | 5 | 2.30% | 7 | | 5.70% | 0.026 | |  |
|  | Neither agree nor disagree | 20 | 18.00% | 17 | 7.90% | 17 | | 13.80% |  |  |  |
|  | Strongly agree | 88 | 79.30% | 193 | 89.80% | 99 | | 80.50% |  |  |  |
| Adequate resources were committed to the implementation | Strongly disagree | 3 | 2.70% | 5 | 2.30% | 6 | | 5.00% | 0.426 | |  |
|  | Neither agree nor disagree | 14 | 12.70% | 19 | 8.80% | 15 | | 12.50% |  |  |  |
|  | Strongly agree | 93 | 84.50% | 191 | 88.80% | 99 | | 82.50% |  |  |  |
| What is the main device you use to access the EHR? | Desktop Computer | 111 | 91.00% | 206 | 93.60% | 105 | | 84.70% | <0.001 | |  |
|  | Laptop | 37 | 30.30% | 38 | 17.30% | 65 | | 52.40% |  |  |  |
|  | Tablet | 0 | 0.00% | 0 | 0.00% | 11 | | 8.90% |  |  |  |
|  | Phone | 5 | 4.10% | 1 | 0.50% | 10 | | 8.10% |  |  |  |
| To see patient test results | Never | 101 | 89.40% | 206 | 94.50% | 107 | | 87.00% | 0.116 | |  |
|  | Occasionally | 8 | 7.10% | 6 | 2.80% | 10 | | 8.10% |  |  |  |
|  | Frequently / Always | 4 | 3.50% | 6 | 2.80% | 6 | | 4.90% |  |  |  |
| To obtain contact information for a patient | Never | 101 | 90.20% | 207 | 94.50% | 105 | | 86.10% | 0.077 | |  |
|  | Occasionally | 6 | 5.40% | 7 | 3.20% | 12 | | 9.80% |  |  |  |
|  | Frequently / Always | 5 | 4.50% | 5 | 2.30% | 5 | | 4.10% |  |  |  |
| To document something in the patient medical record | Never | 98 | 88.30% | 198 | 90.40% | 107 | | 87.00% | 0.726 | |  |
|  | Occasionally | 8 | 7.20% | 16 | 7.30% | 11 | | 8.90% |  |  |  |
|  | Frequently / Always | 5 | 4.50% | 5 | 2.30% | 5 | | 4.10% |  |  |  |
| To identify patients with gaps in their medical care | Never | 101 | 90.20% | 206 | 94.50% | 105 | | 86.80% | 0.076 | |  |
|  | Occasionally | 7 | 6.20% | 6 | 2.80% | 12 | | 9.90% |  |  |  |
|  | Frequently / Always | 4 | 3.60% | 6 | 2.80% | 4 | | 3.30% |  |  |  |
| Do you use paper records for any other reason apart from mentioned above? | Yes | 15 | 13.40% | 10 | 4.70% | 19 | | 16.00% | 0.001 | |  |
|  | No | 97 | 86.60% | 204 | 95.30% | 100 | | 84.00% |  |  |  |
| **EHR IMPACT** | | | | | | | | | | | |
| Costs to my practice in providing care | Highly detrimental / detrimental | 4 | 4.10% | 17 | 8.10% | 5 | | 4.10% | <0.001 | |  |
|  | Neither detrimental nor beneficial | 3 | 3.10% | 7 | 3.30% | 20 | | 16.40% |  |  |  |
|  | Highly beneficial / beneficial | 90 | 92.80% | 187 | 88.60% | 97 | | 79.50% |  |  |  |
| Autonomy of healthcare providers | Highly detrimental / detrimental | 0 | 0.00% | 9 | 4.20% | 7 | | 5.70% | 0.077 | |  |
|  | Neither detrimental nor beneficial | 9 | 8.90% | 20 | 9.30% | 16 | | 13.10% |  |  |  |
|  | Highly beneficial / beneficial | 92 | 91.10% | 187 | 86.60% | 99 | | 81.10% |  |  |  |
| Quality of health care | Highly detrimental / detrimental | 1 | 1.00% | 0 | 0.00% | 1 | | 0.80% | 0.001 | |  |
|  | Neither detrimental nor beneficial | 6 | 5.90% | 2 | 0.90% | 10 | | 8.20% |  |  |  |
|  | Highly beneficial / beneficial | 95 | 93.10% | 214 | 99.10% | 111 | | 91.00% |  |  |  |
| Interactions within the healthcare team | Highly detrimental / detrimental | 5 | 4.90% | 14 | 6.40% | 14 | | 11.50% | 0.026 | |  |
|  | Neither detrimental nor beneficial | 9 | 8.80% | 27 | 12.40% | 24 | | 19.70% |  |  |  |
|  | Highly beneficial / beneficial | 88 | 86.30% | 177 | 81.20% | 84 | | 68.90% |  |  |  |
| Enjoyment of clinical practice | Highly detrimental / detrimental | 3 | 2.90% | 14 | 6.40% | 14 | | 11.50% | 0.030 | |  |
|  | Neither detrimental nor beneficial | 11 | 10.80% | 23 | 10.60% | 21 | | 17.20% |  |  |  |
|  | Highly beneficial / beneficial | 88 | 86.30% | 181 | 83.00% | 87 | | 71.30% |  |  |  |
| Stress levels of healthcare providers | Highly detrimental / detrimental | 8 | 7.80% | 23 | 10.50% | 23 | | 18.90% | 0.003 | |  |
|  | Neither detrimental nor beneficial | 9 | 8.80% | 25 | 11.40% | 24 | | 19.70% |  |  |  |
|  | Highly beneficial / beneficial | 85 | 83.30% | 171 | 78.10% | 75 | | 61.50% |  |  |  |
| Healthcare providers self-image | Highly detrimental / detrimental | 5 | 4.90% | 25 | 11.40% | 15 | | 12.50% | 0.001 | |  |
|  | Neither detrimental nor beneficial | 9 | 8.80% | 24 | 11.00% | 28 | | 23.30% |  |  |  |
|  | Highly beneficial / beneficial | 88 | 86.30% | 170 | 77.60% | 77 | | 64.20% |  |  |  |
| Humaneness of clinical practice | Highly detrimental / detrimental | 4 | 3.90% | 22 | 10.00% | 18 | | 14.80% | 0.002 | |  |
|  | Neither detrimental nor beneficial | 13 | 12.70% | 30 | 13.70% | 29 | | 23.80% |  |  |  |
|  | Highly beneficial / beneficial | 85 | 83.30% | 167 | 76.30% | 75 | | 61.50% |  |  |  |
| The rapport between healthcare providers and patients | Highly detrimental / detrimental | 6 | 5.90% | 20 | 9.20% | 15 | | 12.30% | 0.029 | |  |
|  | Neither detrimental nor beneficial | 11 | 10.80% | 25 | 11.50% | 26 | | 21.30% |  |  |  |
|  | Highly beneficial / beneficial | 85 | 83.30% | 172 | 79.30% | 81 | | 66.40% |  |  |  |
| Personal and professional privacy | Highly detrimental / detrimental | 3 | 2.90% | 9 | 4.10% | 13 | | 10.70% | 0.021 | |  |
|  | Neither detrimental nor beneficial | 8 | 7.80% | 16 | 7.30% | 16 | | 13.10% |  |  |  |
|  | Highly beneficial / beneficial | 91 | 89.20% | 194 | 88.60% | 93 | | 76.20% |  |  |  |
| Healthcare providers access to up to-date information | Highly detrimental / detrimental | 2 | 2.00% | 3 | 1.40% | 5 | | 4.10% | 0.122 | |  |
|  | Neither detrimental nor beneficial | 4 | 3.90% | 13 | 5.90% | 13 | | 10.70% |  |  |  |
|  | Highly beneficial / beneficial | 96 | 94.10% | 203 | 92.70% | 104 | | 85.20% |  |  |  |
| Patients satisfaction with the quality of care they receive | Highly detrimental / detrimental | 3 | 2.90% | 4 | 1.80% | 2 | | 1.70% | 0.037 | |  |
|  | Neither detrimental nor beneficial | 10 | 9.80% | 15 | 6.90% | 21 | | 17.50% |  |  |  |
|  | Highly beneficial / beneficial | 89 | 87.30% | 199 | 91.30% | 97 | | 80.80% |  |  |  |
| Generalists ability to manage more complex problems | Highly detrimental / detrimental | 2 | 2.00% | 4 | 1.80% | 4 | | 3.30% | 0.060 | |  |
|  | Neither detrimental nor beneficial | 9 | 8.90% | 17 | 7.80% | 21 | | 17.40% |  |  |  |
|  | Highly beneficial / beneficial | 90 | 89.10% | 198 | 90.40% | 96 | | 79.30% |  |  |  |
| Comprehensiveness of patient care | Highly detrimental / detrimental | 4 | 3.90% | 3 | 1.40% | 4 | | 3.30% | 0.211 | |  |
|  | Neither detrimental nor beneficial | 6 | 5.90% | 11 | 5.00% | 12 | | 9.80% |  |  |  |
|  | Highly beneficial / beneficial | 92 | 90.20% | 205 | 93.60% | 106 | | 86.90% |  |  |  |
| Efficiency of clinical practice | Highly detrimental / detrimental | 2 | 2.00% | 3 | 1.40% | 5 | | 4.10% | 0.217 | |  |
|  | Neither detrimental nor beneficial | 9 | 8.80% | 9 | 4.10% | 6 | | 4.90% |  |  |  |
|  | Highly beneficial / beneficial | 91 | 89.20% | 207 | 94.50% | 111 | | 91.00% |  |  |  |
| Avoiding errors (such as overlooking drug interactions) | Highly detrimental / detrimental | 4 | 3.90% | 11 | 5.00% | 4 | | 3.30% | 0.386 | |  |
|  | Neither detrimental nor beneficial | 5 | 4.90% | 12 | 5.50% | 13 | | 10.70% |  |  |  |
|  | Highly beneficial / beneficial | 93 | 91.20% | 196 | 89.50% | 105 | | 86.10% |  |  |  |
| **EHR FUNCTIONALITY** | | | | | | | | | | | |
| Obtain and review patient information and data | Very difficult / Somewhat difficult | 4 | 3.90% | 0 | 0.00% | 3 | | 2.50% | 0.017 | |  |
|  | No difference | 3 | 2.90% | 3 | 1.40% | 1 | | 0.80% |  |  |  |
|  | Very easy / Somewhat easy | 95 | 93.10% | 214 | 98.60% | 115 | | 96.60% |  |  |  |
| Document care for my patients | Very difficult / Somewhat difficult | 3 | 3.10% | 0 | 0.00% | 4 | | 3.40% | 0.011 | |  |
|  | No difference | 1 | 1.00% | 1 | 0.50% | 0 | | 0.00% |  |  |  |
|  | Very easy / Somewhat easy | 93 | 95.90% | 216 | 99.50% | 114 | | 96.60% |  |  |  |
| View lab results for my patients | Very difficult / Somewhat difficult | 2 | 2.10% | 0 | 0.00% | 2 | | 1.70% | 0.078 | |  |
|  | No difference | 2 | 2.10% | 1 | 0.50% | 1 | | 0.80% |  |  |  |
|  | Very easy / Somewhat easy | 93 | 95.90% | 215 | 99.50% | 116 | | 97.50% |  |  |  |
| View radiology results for my patients | Very difficult / Somewhat difficult | 2 | 2.10% | 0 | 0.00% | 2 | | 1.70% | 0.014 | |  |
|  | No difference | 4 | 4.20% | 1 | 0.50% | 2 | | 1.70% |  |  |  |
|  | Very easy / Somewhat easy | 90 | 93.80% | 215 | 99.50% | 114 | | 96.60% |  |  |  |
| Prevent adverse events (e.g, drug-drug interaction, drug-allergy interaction) | Very difficult / Somewhat difficult | 4 | 4.10% | 1 | 0.50% | 3 | | 2.50% | 0.017 | |  |
|  | No difference | 2 | 2.10% | 2 | 1.00% | 6 | | 5.00% |  |  |  |
|  | Very easy / Somewhat easy | 91 | 93.80% | 207 | 98.60% | 110 | | 92.40% |  |  |  |
| Track preventive care for my patients | Very difficult / Somewhat difficult | 1 | 1.00% | 1 | 0.50% | 4 | | 3.40% | <0.001 | |  |
|  | No difference | 5 | 5.20% | 1 | 0.50% | 13 | | 11.20% |  |  |  |
|  | Very easy / Somewhat easy | 91 | 93.80% | 213 | 99.10% | 99 | | 85.30% |  |  |  |
| Manage chronic disease conditions for my patients | Very difficult / Somewhat difficult | 1 | 1.00% | 0 | 0.00% | 3 | | 2.50% | 0.006 | |  |
|  | No difference | 3 | 3.10% | 6 | 2.80% | 11 | | 9.30% |  |  |  |
|  | Very easy / Somewhat easy | 92 | 95.80% | 209 | 97.20% | 104 | | 88.10% |  |  |  |
| Manage orders | Very difficult / Somewhat difficult | 1 | 1.00% | 0 | 0.00% | 8 | | 6.80% | <0.001 | |  |
|  | No difference | 4 | 4.20% | 0 | 0.00% | 2 | | 1.70% |  |  |  |
|  | Very easy / Somewhat easy | 91 | 94.80% | 216 | 100.00% | 108 | | 91.50% |  |  |  |
| Manage referrals | Very difficult / Somewhat difficult | 2 | 2.10% | 0 | 0.00% | 5 | | 4.30% | 0.004 | |  |
|  | No difference | 2 | 2.10% | 2 | 0.90% | 5 | | 4.30% |  |  |  |
|  | Very easy / Somewhat easy | 92 | 95.80% | 213 | 99.10% | 107 | | 91.50% |  |  |  |
| Provide patient educational materials | Very difficult / Somewhat difficult | 2 | 2.10% | 0 | 0.00% | 6 | | 5.20% | <0.001 | |  |
|  | No difference | 3 | 3.20% | 5 | 2.30% | 17 | | 14.80% |  |  |  |
|  | Very easy / Somewhat easy | 90 | 94.70% | 208 | 97.70% | 92 | | 80.00% |  |  |  |
| Analyze outcomes of care | Very difficult / Somewhat difficult | 2 | 2.10% | 0 | 0.00% | 5 | | 4.30% | <0.001 | |  |
|  | No difference | 3 | 3.20% | 0 | 0.00% | 10 | | 8.70% |  |  |  |
|  | Very easy / Somewhat easy | 90 | 94.70% | 216 | 100.00% | 100 | | 87.00% |  |  |  |
| Access the EHR from offsite locations | Very difficult / Somewhat difficult | 2 | 2.10% | 2 | 0.90% | 2 | | 1.70% | 0.354 | |  |
|  | No difference | 2 | 2.10% | 1 | 0.50% | 0 | | 0.00% |  |  |  |
|  | Very easy / Somewhat easy | 90 | 95.70% | 209 | 98.60% | 116 | | 98.30% |  |  |  |
| Communicate with my colleagues to coordinate care | Very difficult / Somewhat difficult | 1 | 1.00% | 3 | 1.40% | 6 | | 5.10% | 0.199 | |  |
|  | No difference | 6 | 6.20% | 8 | 3.70% | 6 | | 5.10% |  |  |  |
|  | Very easy / Somewhat easy | 90 | 92.80% | 203 | 94.90% | 105 | | 89.70% |  |  |  |
| Communicate with my patients | Very difficult / Somewhat difficult | 1 | 1.10% | 3 | 1.40% | 6 | | 5.50% | 0.078 | |  |
|  | No difference | 5 | 5.30% | 10 | 4.70% | 10 | | 9.20% |  |  |  |
|  | Very easy / Somewhat easy | 89 | 93.70% | 200 | 93.90% | 93 | | 85.30% |  |  |  |
| Enhance the continuity of care the hospital can provide | Very difficult / Somewhat difficult | 1 | 1.00% | 3 | 1.40% | 3 | | 2.60% | 0.116 | |  |
|  | No difference | 3 | 3.10% | 2 | 0.90% | 6 | | 5.20% |  |  |  |
|  | Very easy / Somewhat easy | 92 | 95.80% | 208 | 97.70% | 106 | | 92.20% |  |  |  |
| Are there any functionalities missing in this EHR? | No | 99 | 95.20% | 210 | 97.70% | 98 | | 81.70% | <0.001 | |  |
|  | Yes | 5 | 4.80% | 5 | 2.30% | 22 | | 18.30% |  |  |  |
| Is there any additional help you would need to use the EHR? | No | 98 | 93.30% | 212 | 98.10% | 111 | | 92.50% | 0.020 | |  |
|  | Yes | 7 | 6.70% | 4 | 1.90% | 9 | | 7.50% |  |  |  |
| **OVERALL EVALUATION OF THE EHR** | | | | | | | | | | | |
| To me, use of this EHR is easy | Strongly disagree | 3 | 2.70% | 0 | 0.00% | 3 | | 2.60% | 0.079 | |  |
|  | Neither agree nor disagree | 7 | 6.30% | 9 | 4.10% | 5 | | 4.30% |  |  |  |
|  | Strongly agree | 101 | 91.00% | 209 | 95.90% | 109 | | 93.20% |  |  |  |
| The EHR screens are user friendly | Strongly disagree | 6 | 5.30% | 1 | 0.50% | 5 | | 4.20% | 0.011 | |  |
|  | Neither agree nor disagree | 9 | 8.00% | 8 | 3.70% | 5 | | 4.20% |  |  |  |
|  | Strongly agree | 98 | 86.70% | 208 | 95.90% | 109 | | 91.60% |  |  |  |
| This EHR provides all functionalities that I expect | Strongly disagree | 5 | 4.40% | 1 | 0.50% | 8 | | 6.80% | 0.001 | |  |
|  | Neither agree nor disagree | 14 | 12.40% | 12 | 5.50% | 5 | | 4.30% |  |  |  |
|  | Strongly agree | 94 | 83.20% | 206 | 94.10% | 104 | | 88.90% |  |  |  |
| Overall, I am satisfied with my experience with this EHR | Strongly disagree | 7 | 6.10% | 0 | 0.00% | 4 | | 3.40% | <0.001 | |  |
|  | Neither agree nor disagree | 21 | 18.40% | 16 | 7.30% | 7 | | 5.90% |  |  |  |
|  | Strongly agree | 86 | 75.40% | 203 | 92.70% | 108 | | 90.80% |  |  |  |
| I would recommend this EHR to other similar practices | Strongly disagree | 5 | 4.40% | 4 | 1.80% | 6 | | 5.00% | 0.001 | |  |
|  | Neither agree nor disagree | 29 | 25.70% | 26 | 12.00% | 11 | | 9.20% |  |  |  |
|  | Strongly agree | 79 | 69.90% | 187 | 86.20% | 102 | | 85.70% |  |  |  |
| My colleagues have negative opinions about this EHR | Strongly disagree | 59 | 51.80% | 143 | 65.30% | 67 | | 56.30% | 0.009 | |  |
|  | Neither agree nor disagree | 37 | 32.50% | 62 | 28.30% | 32 | | 26.90% |  |  |  |
|  | Strongly agree | 18 | 15.80% | 14 | 6.40% | 20 | | 16.80% |  |  |  |
| Use of this EHR interferes with my work | Strongly disagree | 84 | 73.70% | 198 | 90.80% | 99 | | 83.20% | 0.001 | |  |
|  | Neither agree nor disagree | 9 | 7.90% | 6 | 2.80% | 10 | | 8.40% |  |  |  |
|  | Strongly agree | 21 | 18.40% | 14 | 6.40% | 10 | | 8.40% |  |  |  |
| I would be in favor of ceasing use of this EHR in our practice | Strongly disagree | 84 | 75.70% | 201 | 91.40% | 105 | | 88.20% | 0.003 | |  |
|  | Neither agree nor disagree | 6 | 5.40% | 4 | 1.80% | 4 | | 3.40% |  |  |  |
|  | Strongly agree | 21 | 18.90% | 15 | 6.80% | 10 | | 8.40% |  |  |  |
| Use of this EHR requires me to do more work compared to what I used to do | Strongly disagree | 81 | 71.10% | 200 | 90.90% | 92 | | 77.30% | <0.001 | |  |
|  | Neither agree nor disagree | 9 | 7.90% | 3 | 1.40% | 10 | | 8.40% |  |  |  |
|  | Strongly agree | 24 | 21.10% | 17 | 7.70% | 17 | | 14.30% |  |  |  |

**Supplementary Table 1: Differences of EHR experiences among different health care categories.**
