# Supplementary material for: End user experiences of an electronic health records platform in a tertiary hospital system in Kenya
Source: PLoS One. 2025 Sep 19;20(9):e0332249. doi: 10.1371/journal.pone.0332249 (PMC12449015; doi:10.1371/journal.pone.0332249)
Supplement: Attachment — Submitted filename: EHR PLOS ONE - Response Feedback.docx [file pone.0332249.s003.docx]

**END USER EXPERIENCES OF AN ELECTRONIC HEALTH RECORDS PLATFORM IN A TERTIARY HOSPITAL SYSTEM IN KENYAPLOS ONE**

**[PONE-D-25-30375]**

We thank the reviewers for pointing out the comments below and we have tried to address the comments and strengthen the manuscript.

**Response to Comments**

**Overall Editor Comments**

Please ensure that your manuscript meets PLOS ONE's style requirements, including those for file naming.

***Response:***

1. Please ensure that you have specified a) Did participants provide their written or verbal informed consent to participate in this study? b) If consent was verbal, please explain i) why written consent was not obtained, ii) how you documented participant consent, and iii) whether the ethics committees/IRB approved this consent procedure."

***Response:*** Participants provided written informed consent electronically prior to their enrollment in the study. This consent procedure, including the use of electronic consent, was reviewed and approved by the Aga Khan University, Institutional Scientific Ethics and Review Committee (AKU-ISERC).

2. In consent please state in Ethics Method section and manuscript if it is written or verbal. If consent was verbal, please explain a) why written consent was not obtained, b) how you documented participant consent, and c) whether the ethics committees/IRB approved this consent procedure.

***Response:*** Participants provided written informed consent electronically prior to their enrollment in the study. This consent procedure, including the use of electronic consent, was reviewed and approved by the Aga Khan University, Institutional Scientific Ethics and Review Committee (AKU-ISERC).

3. Please include a complete copy of PLOS’ questionnaire on inclusivity in global research in your revised manuscript. Our policy for research in this area aims to improve transparency in the reporting of research performed outside of researchers’ own country or community. The policy applies to researchers who have travelled to a different country to conduct research, research with Indigenous populations or their lands, and research on cultural artefacts.

***Response:*** We have included a copy of the PLOS’ questionnaire on inclusivity in global research.

4. Your ethics statement should only appear in the Methods section of your manuscript. If your ethics statement is written in any section besides the Methods, please delete it from any other section.

***Response:*** We have deleted the Ethics statement in declarations. It only now appears in the methods.

5. Please include captions for your Supporting Information files at the end of your manuscript, and update any in-text citations to match accordingly. Please see our Supporting Information guidelines for more information:

http://journals.plos.org/plosone/s/supporting-information.

***Response:*** We have included captions at the end of the manuscript.

**Reviewer #1:**

6. As such, I am generally supportive of publication with a few minor but essential comments. While the authors use conventional surveys, there have been prior studies using machine learning for healthcare, which should be noted: Environ. Sci. Technol. Lett. 2023, 10, 1017–1022 and Environ. Sci. Technol. 2019, 53, 13970–13980. Specifically, these prior studies have shown that advanced machine learning approaches can provide more details into the underlying mechanisms of biological activity that can complement the experiments. I am not asking the authors to carry out new machine learning calculations at all, but these prior treatments should be noted since these machine learning techniques are well known and a mature field now.

***Response:*** We thank the reviewer for their insightful comment and for highlighting the referenced studies on the use of machine learning (Environ. Sci. Technol. Lett. 2023, 10, 1017–1022 and Environ. Sci. Technol. 2019, 53, 13970–13980). We agree that machine learning is a powerful and mature tool in biomedical research and acknowledge the value it brings in elucidating biological mechanisms. However, we note that the focus of our current study is distinct, as it centers on assessing end-user experiences of an electronic health records platform in a tertiary hospital setting in Kenya. Our methodological approach, which is based on conventional surveys, is designed to capture usability, satisfaction, and contextual factors influencing EHR adoption and interaction from the perspective of frontline healthcare providers.

While machine learning may offer valuable insights in other biomedical domains, particularly in bioactivity prediction and systems biology, it falls outside the immediate scope and objectives of our study. We appreciate the broader context raised by the reviewer and have included a brief note in the discussion to acknowledge the growing role of advanced analytical techniques, including machine learning, in complementary areas of health systems research.

Reviewer #2:

This study addresses a critical area and may serve as a valuable guide for implementing EHR systems in hospital settings, especially in regions with comparable infrastructures and constraints.

Introduction

7. Please spell out the abbreviation "ICT" on first use. Include more details about the EHR implementation strategy. For instance: What types of experts were included on the ICT team? What were the specific roles of clinical informatics team members? How frequently were town halls held, in which format (online or offline), and what topics or updates were covered during these sessions?

***Response:*** We have revised the introduction and included more on the implementation strategy as suggested in the comments.

Methods

8. Please provide a reference for the Michigan Public Health Institute EHR End-User Survey.

***Response:*** We have included the reference for the survey.

9. A large number of statistical tests were conducted, which raises the risk of Type I error. Please apply an appropriate correction method (e.g., Bonferroni or Benjamini-Hochberg) to control for multiple comparisons.

***Response:*** We have added the adjusted p values using Bonferroni correction.

10. The rationale for comparing outcomes by gender is unclear, and these findings were not discussed. I recommend removing this comparison and focusing on comparisons across different professions.

***Response:*** We have removed the table on gender and revised accordingly.

Results

11. Please provide summary tables for EHR impact, functionality, and overall evaluation within the main text.

Clearly reference any supplementary tables when discussing results in the text.

For supplementary materials, consider separating topics into individual tables (e.g., one table for experience, another for environment, etc.).

***Response:*** We have revised the results and added the summary tables in the main manuscript.

12. Review number rounding throughout the manuscript. For example, 78.99% should be rounded to 79.0%, not 78.9%.

***Response:*** We have reviewed all tables and, in the percentages, we have rounded to one decimal point.

Discussion

13. Please include potential solutions or recommendations to address physicians’ concerns—particularly regarding lower satisfaction related to productivity and administrative burden.

***Response:*** We have provided some recommendations in the discussion.
